# Powdery Mildews Are Characterized by Contracted Carbohydrate Metabolism and Diverse Effectors to Adapt to Obligate Biotrophic Lifestyle

**DOI:** 10.3389/fmicb.2018.03160

**Published:** 2018-12-18

**Authors:** Peng Liang, Songyu Liu, Feng Xu, Shuqin Jiang, Jun Yan, Qiguang He, Wenbo Liu, Chunhua Lin, Fucong Zheng, Xiangfeng Wang, Weiguo Miao

**Affiliations:** ^1^College of Plant Protection, Hainan University, Haikou, China; ^2^Key Laboratory of Green Prevention and Control of Tropical Plant Diseases and Pests (Hainan University), Ministry of Education, Haikou, China; ^3^Department of Crop Genomics and Bioinformatics, College of Agronomy and Biotechnology, National Maize Improvement Center of China, China Agricultural University, Beijing, China; ^4^State Key Laboratory of Agrobiotechnology, College of Biological Sciences, China Agricultural University, Beijing, China

**Keywords:** *Erysiphales*, *Oidium heveae*, genome, gene family contraction, fatty acids, CSEPs, positive selection, adaptive evolution

## Abstract

Powdery mildew is a widespread plant disease caused by obligate biotrophic fungal pathogens involving species-specific interactions between host and parasite. To gain genomic insights into the underlying obligate biotrophic mechanisms, we analyzed 15 microbial genomes covering powdery and downy mildews and rusts. We observed a genome-wide, massive contraction of multiple gene families in powdery mildews, such as enzymes in the carbohydrate metabolism pathway, when compared with *ascomycete* phytopathogens, while the fatty acid metabolism pathway maintained its integrity. We also observed significant differences in candidate secreted effector protein (CSEP) families between monocot and dicot powdery mildews, perhaps due to different selection forces. While CSEPs in monocot mildews are likely subject to positive selection causing rapid expansion, CSEP families in dicot mildews are shrinking under strong purifying selection. Our results not only illustrate obligate biotrophic mechanisms of powdery mildews driven by gene family evolution in nutrient metabolism, but also demonstrate how the divergence of CSEPs between monocot and dicot lineages might contribute to species-specific adaption.

## Introduction

Obligate biotrophic fungal pathogens, which cause powdery mildews, downy mildews and rusts, are noted for their narrow host ranges and absolute dependence on living plant cells (Spanu et al., 2010). Although these widespread pathogens do not kill their hosts, they nonetheless cause devastating damage to plants. In the ecosystem, eukaryotic fungal and oomycotal microbes play extremely important roles in plant–microbe interactions and may include saprotrophs, necrotrophs, hemibiotrophs, and obligate biotrophs. Comparisons of different microbial genomes highlight striking features shared by obligate biotrophic pathogens, including genome size expansion and gene losses in secondary metabolism (Spanu, 2012). Additionally, obligate biotrophs all form morphologically invasive haustoria for nutrient uptake. These similarities are likely indicators of convergent evolution among obligate biotrophic pathogens enabling them to inhabit a common ecological niche (Bindschedler et al., 2016). Recently, genomic analyses of powdery mildew species have provided information about their evolution and pathogenicity (Hacquard et al., 2013; Menardo et al., 2016, 2017a,b; Frantzeskakis et al., 2018; Müller et al., 2018; Wu et al., 2018). However, for reasons such as an inefficient transgenic pipeline, the molecular mechanism underpinning the obligate biotrophic lifestyle remains unknown.

There are more than 400 species of powdery mildews that are able to colonize nearly 10,000 plant species (Takamatsu, 2004) and they exhibit a wide range of host specificities. Some powdery mildews like *Golovinomyces cichoracearum* affect many dicot plants, including tobacco, cucurbit, and *Arabidopsis thaliana*, while others, such as the formae speciales *hordei* and *tritici* of *Blumeria graminis*, only infect the monocots barley and wheat, respectively (Dean et al., 2012). The zigzag model illustrates the coevolution between pathogenicity-related genes or effectors and host resistance genes (Jones and Dangl, 2006). Moreover, powdery mildews harbor a diverse superfamily of species-specific candidate secreted effector proteins (CSEPs) (Pedersen et al., 2012; Hacquard et al., 2013; Wicker et al., 2013; Jones L. et al., 2014; Menardo et al., 2017a; Frantzeskakis et al., 2018; Müller et al., 2018) that are instrumental in modulating host immunity and disease resistance (Pedersen et al., 2012). Although researchers have found that effector repertoires were influenced by host resistance (O’Connell et al., 2012; Jones L. et al., 2014), further studies are needed to better understand the arms race between powdery mildews and their hosts.

These attributes, including abundant pathogenicity and genomic variability, make powdery mildew an ideal model for understanding the complexity of obligate biotrophs. In this study, we sequenced the genome of a powdery mildew (*Oidium heveae*) from the rubber tree (*Hevea brasiliensis*) and applied a comparative genomics approach to investigate the striking variations among microorganisms with differing lifestyles. We also performed positive selection detection to account for CSEP rapid evolution. The aim of this study is to elucidate genomic adaptive evolution patterns involved in nutrient metabolism adaptation and the divergence of CSEPs.

## Results

### Assembly and Annotation of the *Oidium heveae* Genome

Once *Oidium heveae* conidia land on immature leaves of the rubber tree (*Hevea brasiliensis*), they germinate and form appressoria. From there, pathogens produce penetration structures and hyphae within 24 h post-inoculation, then produce disease symptoms including defoliation and curling of leaves, growth retardation (Supplementary Figure S1), and latex yield reduction (Limkaisang et al., 2005). After we isolated *O. heveae* strain HO-73 from the rubber tree cultivar *H. brasiliensis* Reyan 7-33-97, we sequenced the *O. heveae* genome using the Illumina HiSeq platform and generated paired-end reads for *O. heveae*, as well as 15.7 Gbps of high-quality datasets (Supplementary Table S1). *O. heveae*’s genome assembly total length was 62.28 Mb (Table 1) and repeat sequences accounted for an estimated 49.17% of the genome (Supplementary Table S2). Because of its highly repetitive nature, the genome was estimated to be approximately 110.57 Mb (Supplementary Figure S2), corresponding to a 142.2-fold coverage of the *O. heveae* genome. The final genome assembly contained 13,271 scaffolds (N50 = 51.94 kb) and 97.18% of the eukaryotic core genes were mapped in full-length using CEGMA (Supplementary Table S3).

**Table 1:** Summary of genome assembly and annotation for *Oidium heveae*.

Assembly	
Genome-sequencing depth	142.2
Estimated genome size (Mb)	110.57
No. of scaffolds	13,271
Total length of scaffolds (bp)	62,280,838
N50 of scaffolds (bp)	51,942
Longest scaffolds (bp)	364,184
GC content of the genome (%)	38.43
CEGMA (%)	97.18

**Prediction and annotation**	

No. of predicted protein-coding genes	6,377
Average gene length (bp)	1,411.38
Gene density (gene per kb)	0.1
Percentage of gene length in the genome (%)	14.32
tRNAs	374
rRNAs	13
Repeat sequences (%)	49.17
NCBI non-redundant protein (nr)	5,575
GO	4,728
KEGG	2,551
EuKaryotic Orthologous Groups (KOG)	2,661
CSEPs	133


To improve *de novo* gene prediction, RNA-seq transcriptomes were obtained from two fungal libraries (0 hpi, 30 dpi) and two plant-pathogen mixed libraries (24 hpi, 3 dpi) (Supplementary Table S4). A combination of *ab initio* predictions, assembled RNA-seq transcripts, and homologous proteins identified from *Blumeria graminis* f. sp. *hordei* (*Bgh*), *Blumeria graminis* f. sp. *tritici* (*Bgt*), and *Erysiphe necator* (*En*) led to the annotation of 6,377 high-confidence protein-coding genes, a number that is similar to that of other powdery mildews (Supplementary Table S5), including the dicot powdery mildew *En* and the monocot powdery mildews *Bgh* and *Bgt*. We further computationally identified 133 coding CSEP genes in *O. heveae* (Supplementary Table S5, see the section “Materials and Methods”), which is similar to the number in *En* (149) and other dicot host plant infecting powdery mildews (Jones L. et al., 2014; Wu et al., 2018), but less than in *Bgh* (Pedersen et al., 2012; Frantzeskakis et al., 2018).

### Contraction of Pathogenicity-Related Gene Families in Powdery Mildews

To attain genomic insights into the obligate biotrophic mechanism, we performed a genome-wide comparison among 15 species, including seven obligate biotrophs (*Oidium heveae*, *Erysiphe necator*, *Blumeria graminis* f. sp. *hordei*, *Blumeria graminis* f. sp. *tritici*, *Puccinia graminis* f. sp. *tritici*, *Melampsora laricis-populina*, and *Hyaloperonospora arabidopsidis*), three hemibiotrophs (*Magnaporthe oryzae*, *Colletotrichum higginsianum*, and *Fusarium graminearum*), two necrotrophs (*Sclerotinia sclerotiorum* and *Botrytis cinerea*), and three saprotrophs (*Saccharomyces cerevisiae*, *Aspergillus nidulans*, and *Neurospora crassa*) (Supplementary Table S6). The obligate biotrophic species’ genome sizes ranged from 81.6 to 180 Mbp, while the others ranged from 12.2 to 53.4 Mbp (Supplementary Table S7). Annotated gene numbers for the four powdery mildew species are at the lower end among the 15 genomes, while the other 10 species contain over 10,000 genes, with the exception of *S. cerevisiae*, which contains 6,692 genes (Supplementary Table S7).

**FIGURE 1:**
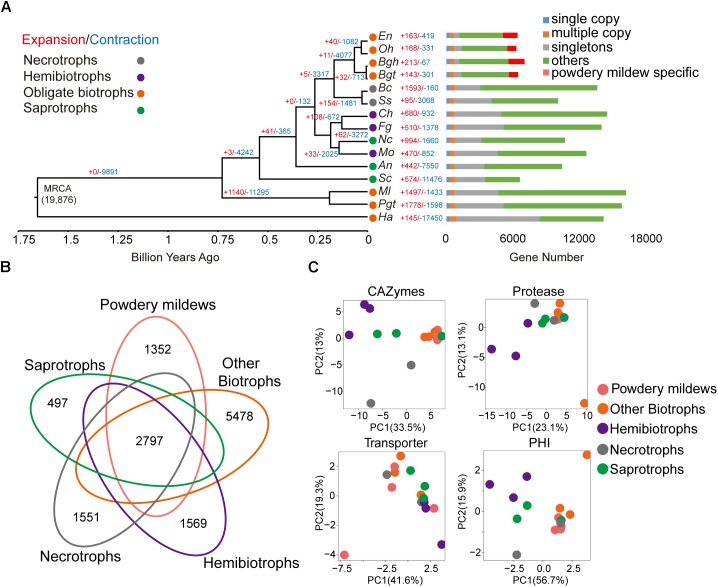
Powdery mildews have contracted gene family sizes and a similar pathogenicity-related repertoire for their obligate biotrophic lifestyle. **(A)** The phylogenetic relationship, expansion, and contraction of gene families among 15 microorganisms. The phylogenetic tree is based on single-copy, orthologous gene families shared by all species. Branch numbers indicate the number of gene families that have expanded (red) and contracted (blue) after the split from the common ancestor. MRCA, most recent common ancestor. Circle colors represent different lifestyles. Time lines indicate divergence times among the species. The bar diagram shows the distribution of genes based on the orthologous gene clusters. The following abbreviations in all figures are used for each species: *Oidium heveae* (*Oh*), *Erysiphe necator* (*En*), *Blumeria graminis* f. sp. *hordei* (*Bgh*), *Blumeria graminis* f. sp. *tritici* (*Bgt*), *Puccinia graminis* f. sp. *tritici* (*Pgt*), *Melampsora laricis-populina* (*Ml*), *Hyaloperonospora arabidopsidis* (*Ha*), *Magnaporthe oryzae* (*Mo*), *Colletotrichum higginsianum* (*Ch*), *Fusarium graminearum* (*Fg*), *Sclerotinia sclerotiorum* (*Ss*), *Botrytis cinerea* (*Bc*), *Saccharomyces cerevisiae* (*Sc*), *Aspergillus nidulans* (*An*), *Neurospora crassa* (*Nc*). **(B)** The Venn diagram shows shared and unique orthologous families among different trophic lifestyles. Gene families from every lifestyle are the sum of the families from species belonging to the same lifestyle. **(C)** Principal component analysis based on CAZymes, protease, transporter, and pathogen-host interaction (PHI) genes for different lifestyles.

A phylogenetic tree was constructed using 287 single-copy orthologous genes extracted from the 15 microorganisms using a maximum-likelihood algorithm, with all of the branches obtaining 100% bootstrap values. As expected, the four powdery mildew species clustered together and far from the other three obligate biotrophic species: *P. graminis* f. sp. *tritici*, *M. laricis-populina*, and *H. arabidopsidis* (Figure 1A). The estimated time of divergence between *Oomycota* and fungi approximately 1.654 billion years ago (Bya) (Figure 1A), followed by *Basidiomycota* and *Ascomycota* approximately 0.729 Bya, suggesting distant relationships among the studied obligate biotrophic pathogens. Furthermore, we predicted divergence between powdery mildews and *Sclerotiniaceae* was approximately 168 million years ago (Mya) and the divergence between monocot and dicot powdery mildews was approximately 66.1 Mya.

Gene family expansion and contraction are common features seen in pathogen evolution. By integrating the genes within the 15 genomes through pairwise protein alignment, we identified a total of 19,876 gene families, comprising 121,493 genes (see the section “Materials and Methods”). All the powdery mildews showed a small number of expanded or contracted families compared with their lineage’s common ancestor. The significantly expanded families (*P* < 0.05) for each powdery mildew are annotated to function in DNA integration, nucleic acid metabolic processes, and phosphorylation (Supplementary Table S8), as well as the species-specific gene families. The powdery mildew lineage showed a contraction of significant gene families. The common ancestor of powdery mildews showed 11 expanded and 4,077 contracted gene families, compared to the common ancestor of *Erysiphales* and *Helotiales*. Interestingly, the contracted gene families were enriched mainly for carbohydrate metabolism, such as carbohydrate metabolic process (GO:0005975, *P* = 2.92 × 10^-8^), carbohydrate transport (GO:0008643, *P* = 8.80 × 10^-10^), and carbohydrate catabolic process (GO:0016052, *P* = 9.43 × 10^-5^) (Supplementary Table S9). Contracted gene families were also significantly enriched for the starch and sucrose metabolism pathway (ID: ko00500, *P* = 3.66 × 10^-4^) and pentose and glucuronate interconversions (ID: ko00040, *P* = 2.89 × 10^-3^), suggesting a decreased demand for carbohydrate metabolism in powdery mildews, compared to hemibiotrophic and necrotrophic phytopathogens.

To explore the pathogenic gene contents of obligate biotrophic genomes, we generated orthologous families from the five groups, including hemibiotrophs, necrotrophs, saprotrophs, other biotrophs minus powdery mildews and powdery mildews, and found only 2,797 conserved orthologous families (Figure 1B). Despite sharing the same lifestyle, the predicted genes, as well as the orthologous gene families, revealed considerable differences among powdery mildews and other obligate biotrophs. We identified CAZymes, protease, transporter, and PHI classifications for the 15 microorganisms (Supplementary Table S10). Fungal pathogens use CAZymes and proteases to degrade host cell walls and tissues for nourishment (Zhao et al., 2013; Xu et al., 2016). Transporters play an essential role in nutrient uptake, but also function in the export of compounds involved in pathogenesis and virulence (Morales-Cruz et al., 2015; Verma et al., 2016). The PHI-base contains experimentally verified pathogenicity, virulence, and effector genes from many plant and animal pathogens (Urban et al., 2017). Despite distant taxonomical relationships, principal component analysis showed that obligate biotrophic species possessed similar repertoires within CAZymes classifications (Figure 1C). The numbers of CAZymes and proteases from powdery mildews and other obligate biotrophs was significantly less than those of hemibiotrophic and necrotrophic phytopathogens (Supplementary Figure S3). In particular, powdery mildews gave up most CAZyme families involved in degrading pectins (Supplementary Figure S4), while hemibiotrophic and necrotrophic phytopathogens have an expanded pectinase family (Zhao et al., 2013). This reduced CAZyme and protease gene content indicate that the need of obligate biotrophs to attack their hosts has decreased.

### Pruned Carbohydrate Metabolism Pathways in Powdery Mildews

To characterize the reduced genes in both pathogenic and nutritional metabolism pathways in powdery mildews, compared with hemibiotrophic and necrotrophic phytopathogens, we systematically searched a core set of 2,646 orthologous families present in *Magnaporthe oryzae*, *Colletotrichum higginsianum*, *Fusarium graminearum*, *Botrytis cinerea* and *Sclerotinia sclerotiorum*, as well as in powdery mildews. Within the 2,646 ascomycete pathogen core families, we confirmed the presence of genes enriched for energy metabolism and primary signaling pathways, similar to previous *Bgh* study (Kusch et al., 2014), such as the citrate (TCA) cycle (ID: ko00020, *P* = 5.61 × 10^-3^) and mitogen-activated protein kinase (MAPK) signal pathway (ID: ko04011, *P* = 8.09 × 10^-6^) (Supplementary Table S11). Interestingly, the lipid metabolism pathway, including glycerophospholipid metabolism (ID: ko00564, *P* = 1.05 × 10^-2^) and glycerolipid metabolism (ID: ko00500, *P* = 4.16 × 10^-3^), was also an important part of the core genes. This means that these lipid metabolism-related and primary signaling pathways are indispensable for both powdery mildews and non-obligate biotrophs.

**FIGURE 2:**
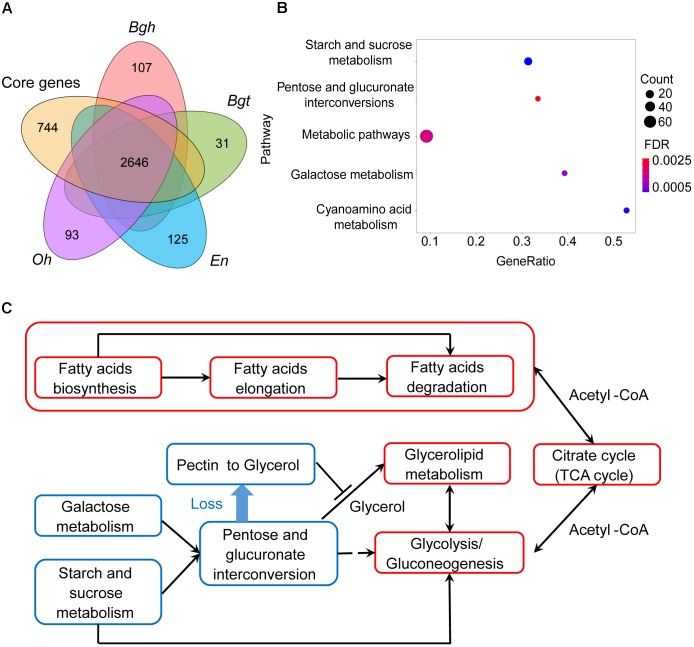
Powdery mildews’ MACGs enrich for the carbohydrate-related metabolism pathway and conserved core genes enrich for the lipid-related metabolism pathway. **(A)** Comparison of orthologous genes between powdery mildews and ascomycete pathogen core genes. The core genes represent the common gene families shared by five *ascomycete* pathogens, including *Magnaporthe oryzae*, *Colletotrichum higginsianum*, *Fusarium graminearum*, *Botrytis cinerea*, and *Sclerotinia sclerotiorum*. Numbers indicate conserved families and species-specific families. Conserved core genes contain 2,646 orthologous families. The MACGs contain 744 orthologous families. **(B)** MACGs enrich for the carbohydrate-related metabolic pathway. The size of the dot indicates the number of genes associated with MACG pathways. Statistical analyses used the false discovery rate (FDR < 0.05). **(C)** Powdery mildews employ carbohydrate metabolism-related pathways and maintained lipid metabolism-related pathways. The red frames indicate integrated pathways. The blue frames indicate contractive pathways. Solid lines indicate direct relationships between pathways. Dashed lines indicate multiple steps in the process.

Next, we identified 744 gene families that are absent in all powdery mildews, which are referred to as missing ascomycete pathogen core genes (MACGs) (Figure 2A, see the section “Materials and Methods”) and are annotated to a diverse set of metabolic pathways. Surprisingly, MACGs are also enriched for carbohydrate metabolism, including starch and sucrose metabolism, galactose metabolism, and the pentose and glucuronate interconversions pathway (Figure 2B). To our knowledge, galacturonate is an important carbon source for fungi living on decaying host plants because it is the principal component of pectin (Hilditch et al., 2007; Zhang et al., 2011). However, genes were absent in the D-galacturonate degradation module in powdery mildews, resulting in their loss of the ability to degrade pectin to glycerol in the pentose and glucuronate interconversions pathway (Figure 2C and Supplementary Figure S5). Consistent with CAZyme clusters and with contracted gene family analysis, MACGs suggest a genetic basis for the decreased ability of powdery mildews to degrade plant cells.

Compared with pruned carbohydrate metabolism pathways, lipid metabolism pathways are relatively complete. Genes encoding enzymes involved in fatty acid biosynthesis and fatty acid elongation were found in powdery mildew genomes (Supplementary Figures S6, S7), as well as in those of the necrotrophic *Botrytis cinerea* and *Magnaporthe oryzae* (Supplementary Table S12). Furthermore, the downstream TCA cycle is also complete (Supplementary Figure S8). These results, together with research on plant fatty acid transfer to parasites (Jiang et al., 2017; Luginbuehl et al., 2017), indicate that a lipid carbon source might be a novel alternative pathway providing energy for parasites (Figure 2C). Our findings indicate that complete fatty acid metabolism as a nutritional source compensates for inefficient plant cell wall degradation via carbohydrate metabolism, a possible ecological adaption of powdery mildews.

### CSEPs Are Likely Subject to Positive Selection

**FIGURE 3:**
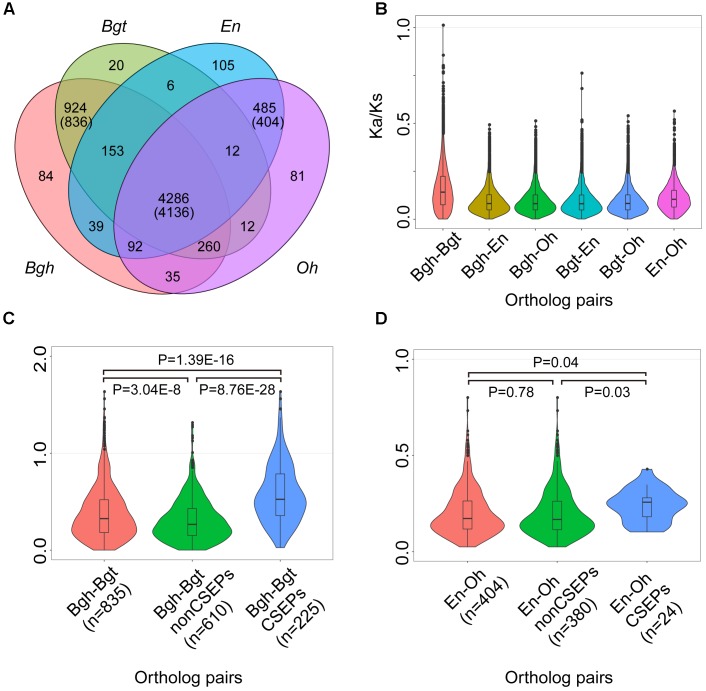
CSEP *K*a/*K*s ratios are higher than conserved and lineage-specific orthologous genes. **(A)** Comparison of orthologous genes among powdery mildews. Numbers in parentheses indicate shared families corresponding to single-copy orthologs. **(B)** Pairwise *K*a/*K*s show purifying selection in 4,136 single-copy ortholog families shared by four powdery mildews. Each family has six combinations (*n* = 4,136 for each combination). **(C)** Pairwise *K*a/*K*s for the 836 single-copy gene families shared by two monocot powdery mildews. After filtering for *K*s > 0 (*n* = 835) for monocot powdery mildews. **(D)** Pairwise *K*a/*K*s for 404 single-copy gene families shared by two dicot powdery mildews. Statistical analyses were conducted using Student’s *t*-test between two groups.

To investigate evolutionary features of the genes shared by powdery mildews, we calculated the ratio of non-synonymous (*K*a) to synonymous (*K*s) substitutions for all of the pairs among the 4,136 single-copy orthologous powdery mildew gene families (Figure 3A). The *K*a/*K*s ratios for the entire set of orthologous genes shared by the four pathogens ranged from 0.001 to 1.012, and only one gene pair exhibited strong signatures associated with positive selection. Most (99.71%) orthologous genes exhibited *K*a/*K*s ratios less than 0.5, indicating that the majority of the orthologous gene pairs are subject to purifying selection (Figure 3B). As a matter of fact, the core single-copy orthologous genes cover a diverse array of functional proteins affecting multiple lipid metabolic processes (Supplementary Table S13). The strong purifying selection signature of the powdery mildews’ conserved genes suggests that lipid metabolism functions are indispensable.

We next computed *K*a/*K*s within orthologous genes shared by monocot and dicot powdery mildews, generating single-copy orthologous groups (836) shared by *Bgh* and *Bgt* (Figure 3A) and single-copy orthologous groups (404) shared by *En* and *Oh* (Figure 3A). Remembering that CSEPs were enriched in the lineage-specific orthologous groups, we used the improved CSEP pipeline (Supplementary Figure S9) and identified 133 CSEPs for *O. heveae* (Supplementary Figure S10), 494 CSEPs for *Bgh* and 421 CSEPs for *Bgt*. In addition, a previous study identified 149 CSEPs for *En*. However, caution is needed when comparing the evolutionary rates among the *Bgh*–*Bgt* subgroups, since the CSEP subgroups showed strikingly higher evolutionary rates compared to genes encoding other proteins, differences supported by statistical analyses (Figure 3C). We proved that among the 225 CSEP groups with *K*a/*K*s ratios ranging from 0.026 to 1.633, 19 CSEP groups showed significant evidence of positive selection. In contrast, the *En-Oh* groups’ CSEP subgroup did not show evidence of positive selection. However, *En-Oh* CSEP *K*a/*K*s ratios also displayed statistically higher evolutionary rates (Figure 3D). Overall, based on pairwise gene analysis of the *K*a/*K*s, we found that CSEPs were under positive selection, while most genes were under strong purifying selection. Furthermore, we observed a lower *K*a/*K*s ratio for CSEPs pairwise between *En* and *Oh* than pairwise between *Bgh* and *Bgt*, suggesting a potentially different evolutionary trace on diverse pathogenicity genes.

### Distinct Characterization of CSEPs Between Monocot and Dicot Powdery Mildews

**FIGURE 4:**
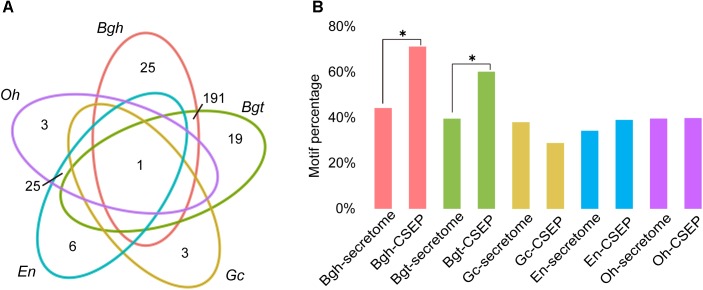
Larger CSEP family sizes and more motifs in monocot powdery mildews compared to dicot powdery mildews. **(A)** Comparison of orthologous CSEP families among powdery mildews. Each number in the diagram represents the number of CSEP families within a group. **(B)** Percentage of [Y/F/W]×C motifs in powdery mildew CSEPs and secretomes except for CSEP. Asterisks indicate significant difference (^∗^*P* < 0.05, Fisher’s exact test).

The function of CSEPs is to subvert innate plant immune systems, thus enabling powdery mildew infection. Wicker et al. (2013) demonstrated that these genes are under selective pressure to evolve rapidly. To gain a better understanding of the difference of CSEP between dicot powdery mildews and monocot powdery mildews, we predicted *Arabidopsis* powdery mildew effectors based on the *Golovinomyces cichoracearum* (*Gc*) race UCSC1 genome^1^. We ultimately identified 97 CSEPs for *Gc*. Interestingly, *Gc* has the fewest CSEPs among the sequenced powdery mildews. Homology-based alignment search and grouping by OrthoMCL returned only one orthologous group shared by all five powdery mildews (Figure 4A), indicating that CSEP genes are highly variable at the amino acid level. Consistent with two previous studies, dicot powdery mildews contained fewer CSEP members than monocot powdery mildews (Jones L. et al., 2014; Wu et al., 2018). There were only 25 CSEP orthologous groups shared by *En* and *Oh*, but *Bgh* and *Bgt* shared 191 groups (Figure 4A and Supplementary Tables S14–S18). Therefore, the difference between monocot and dicot lineage-specific CSEPs accounts for a great difference in CSEP numbers.

In addition to CSEP, fungi also possess effectors that are secreted through the non-classical pathway (without a signal peptide). We also identified non-classically secreted proteins and predicted 1148, 1230, 1225, 1359, and 1191 candidate non-classical effectors for *Bgh*, *Bgt*, *En*, *Gc*, and *Oh*, respectively (Supplementary Table S19). Unlike CSEP, we found no significant quantitative differences among the non-classical effector repertoires of powdery mildews.

Motif-level comparison between monocot and dicot powdery mildews also reveals differences in their [Y/F/W] × C motifs, likely a prevalent structure in CSEPs from cereal powdery mildews (Godfrey et al., 2010). The [Y/F/W] × C motif typically occurs in the N-terminal region after signal peptide cleavage sites, and 72.26% and 60.27% of secreted effectors possess this motif in *Bgh* and *Bgt*, respectively. The predicted CSEPs with [Y/F/W] × C motifs exhibited a significant enrichment (*P* = 4.38 × 10^-10^, *P* = 3.07 × 10^-5^; Fisher’s exact test) when compared to the 44.25% and 39.60% frequencies in the predicted *Bgh* and *Bgt* secretomes minus CSEPs, respectively (Figure 4B). It is worth noting that only 28.87%, 39.08%, and 39.85% of CSEPs associated with this motif were identified in *Gc*, *En* and *Oh*, respectively. Moreover, the [Y/F/W] × C motif was not significantly enriched in the CSEPs of Gc, En, and Oh compared to their respective secretomes minus CSEPs (Figure 4B), suggesting that dicot powdery mildews do not preferentially possess the [Y/F/W] × C motif.

**FIGURE 5:**
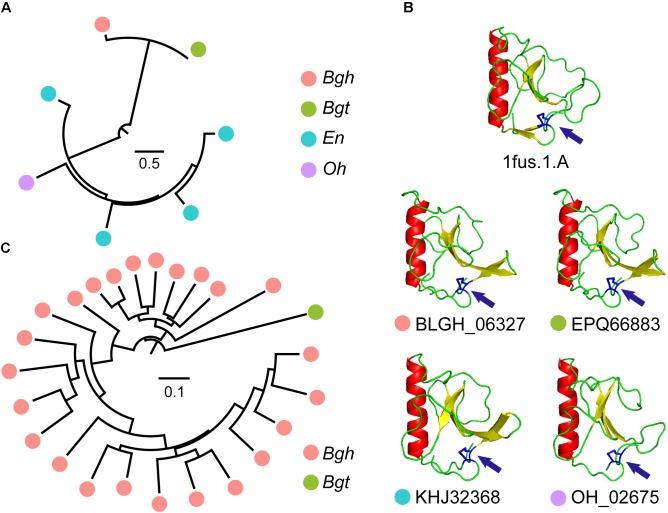
Ribonuclease-CSEP duplicates from a common ancestor and their conserved structure. **(A)** Phylogenetic analysis of OG15 CSEPs reveal gene expansion in dicot powdery mildews. The color of the circles indicates different powdery mildew species. **(B)** Similar ribonuclease 3-dimensional structures for OG15 CSEPs. The 1fus.1.A model is a ribonuclease template. Arrows indicate predicted disulfide bonds between N- and C-terminal cysteines. **(C)** Phylogenetic analysis of OG1 CSEPs reveals duplication in monocot powdery mildews.

In many CSEPs, ribonuclease-like domains can be identified. The InterProScan pipeline identified 73 CSEPs from *Bgh* that showed similarity to ribonuclease domains. Moreover, 31, 14, and 13 CSEPs from *Bgt*, *En*, and *Oh*, respectively, matched ribonuclease domains. In particular, Orthologous Group 15 (OG15), shared by *Bgh*, *Bgt*, *En*, and *Oh*, and Orthologous Group 1 (OG1), shared only by *Bgh* and *Bgt*, are ribonuclease domain-containing gene groups. To our surprise, gene duplication of OG15 occurred in dicot powdery mildews (Figure 5A) and the predicted folds of the CSEPs were highly similar to those of ribonuclease T1 (Figure 5B). We also detected a disulfide bond structure related to the [Y/F/W] × C motif (Figure 5B). We found no positive selection in OG15 using either the site model or the branch-site model in PAML, further strengthening the degree of similarity between these proteins. In contrast to OG15, OG1 possessed a gene duplication event in *Bgh* (Figure 5C). The group contained 24 ribonuclease-like CSEPs, of which 23 belong to *Bgh*. We detected 14 positive selection sites reflecting variation along the *Bgh* branch (Supplementary Table S20 and Supplementary Figure S11). The diverse ribonuclease-like CSEP group, together with the RNase-Like Proteins associated with Haustoria (RALPH) effector-associated results (Spanu, 2017), all suggested that CSEP genes may have a common, ancestral ribonuclease gene that is subject to active duplication.

### CSEPs Are Subject to Positive Selection in Monocot Powdery Mildews but Negative Selection in Dicot Powdery Mildews

**FIGURE 6:**
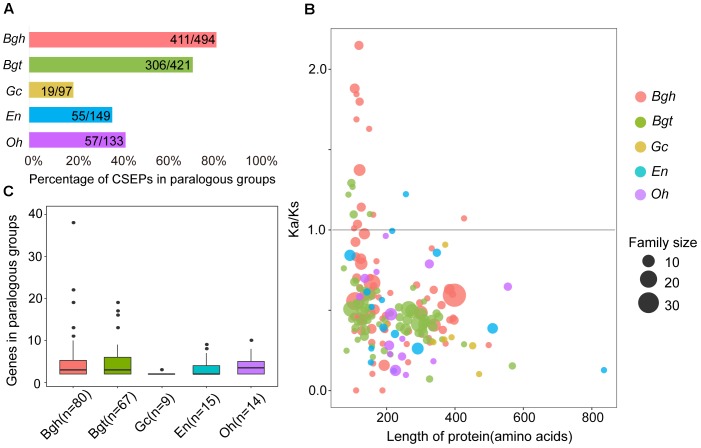
Comparison of CSEP paralogs shows fewer families and purifying selection in dicot powdery mildews and more families and positive selection in monocot powdery mildews. **(A)** Percentage of CSEPs in paralogous families. **(B)** Gene number for CSEP paralogous families. **(C)** *K*a/*K*s within all paralogous CSEP families plotted versus protein length. *K*a/*K*s values were calculated as averages of each pairwise gene in the family. Circles sizes indicate genes numbers for relative families. Circle colors indicate different powdery mildew species.

By counting numbers within each species for paralogous CSEPs, we found a higher percentage of paralogous CSEPs and more genes of paralogous groups for monocot powdery mildews (Figures 6A,B). The percentages of CSEP paralogous groups were 19.59% (*Gc*), 36.91% (*En*) and 42.86% (*Oh*), almost half of the 83.20% (*Bgh*) and 72.68% (*Bgt*) (Figure 6A), in addition, dicot powdery mildews had fewer paralogous groups and average gene members than *Bgh* and *Bgt* (Figure 6B). Furthermore, no *Gc*, *En*, and *Oh* groups contained more than 10 genes, while five groups in *Bgh* and four groups in *Bgt* each contained more than 10 members (Figure 6B), indicating that monocot powdery mildews were subjected to strong CSEP duplication during speciation.

Adaptive divergence at the molecular level may be reflected by an increased *K*a/*K*s rate within genes (Tong et al., 2017). To further elucidate the evolutionary divergence pattern of CSEPs, we tested selection pressure within each group of paralogous pairs using the KaKs_calculator. We analyzed the relationships between CSEP protein lengths and degrees of inferred selection pressure based on the respective average for each group (Figure 6C). Monocot powdery mildews exhibited greater *K*a/*K*s ratios compared to dicots, as 12 groups on *Bgh* and 5 groups on *Bgt* showed signatures of positive selection. In contrast, only one group derived from *En* was subjected to positive selection, and we did not find any evidence of positive selection on any group from *Gc* and *Oh*. Most of the CSEP groups consisted of proteins shorter than 400 amino acids. In particular, we found that positively selected CSEP families are composed of shorter proteins (100–200 amino acids).

We concluded that, based on the selection patterns of CSEPs associated with different powdery mildew lineages, while monocot powdery mildews exhibited positive selective groups with a preference for small groups and shorter proteins, the groups consisting of large groups and longer proteins displayed purifying selection. Dicot powdery mildews exhibited contractive CSEP groups, likely due to strong negative selection. Coincidentally, a recent study also found that dicot powdery mildews tend to practice polyphagy, which might have lessened selective pressure for escalating with a particular host (Wu et al., 2018). The different selection forces in CSEPs between monocot and dicot powdery mildews, as well as the absence of positive *K*a/*K*s ratios from *Gc*, *En*, and *Oh*, may reflect the absence of antagonistic evolution in the dicot hosts, as most of the cultivated grapes and rubber trees fully succumb to powdery mildews (Jones L. et al., 2014; Li et al., 2016).

## Discussion

### Fatty Acids Serve as an Alternative Nutrient Source and May Compensate for Insufficient Carbon-Based Energy Caused by Pruned Carbohydrate Metabolisms

Obligate biotrophy is an extraordinary feature that evolved independently in plant pathogenic fungi and *oomycetes* (Spanu, 2012), yet what underpins the obligate biotrophic lifestyle of powdery mildews remains unclear. To infect a host with the goal of obtaining nutrition, microbes should first produce a variety of proteins, mostly CAZymes, to degrade plant cell walls (O’Connell et al., 2012). Our protein cluster analyses revealed that obligate biotrophic species, especially powdery mildews, exhibited similar repertoires of CAZymes (Figure 1C). Hemibiotrophic and necrotrophic pathogens have expanded CAZyme and protease sets (Supplementary Figure S3 and Supplementary Table S10), compared to significantly reduced gene sets found in obligate biotrophs and mycorrhizal symbionts (Baxter et al., 2010; Amselem et al., 2011; Duplessis et al., 2011; O’Connell et al., 2012; Tisserant et al., 2013). These enzymes can usually generate damage-associated molecular patterns that may trigger host immunity. Thus, the gene loss may be beneficial for the biotrophic lifestyle by reducing opportunities for hosts to elicit rejection (Spanu, 2012).

Biotrophic arbuscular mycorrhizal symbionts generally receive sugar-based carbon from plants (Bago et al., 2002; Trepanier et al., 2005; Jiang et al., 2017). But in recent studies, lipid-based energy transference between host and parasite has been revealed as widespread phenomena in several biotrophic, interorganismic interactions (Caffaro and Boothroyd, 2011; Elwell et al., 2011; Herren et al., 2014; Luginbuehl et al., 2017). Moreover, reports that plant fatty acids can be transferred to powdery mildew and mutualistic mycorrhizal fungi suggest that fatty acids are also an indispensable nutrition source for parasites (Jiang et al., 2017; Luginbuehl et al., 2017). This is also evident given the accumulation of lipids in conidiophores, which supports the interpretation that *B. graminis* uses lipids as a primary energy source (Both et al., 2005). Our genomic analyses also support these previous studies. Pruned gene families and gene loss causing incomplete carbohydrate metabolic pathways (Figures 1, 2) can be a means of eliminating host death by preventing cell wall degradation. At the same time, and to obtain sufficient energy, the parasite maintains integrative, fatty acid metabolism-related pathways and downstream TCA cycles to compensate their deficient carbohydrate metabolisms (Figure 2C and Supplementary Figures S6–S8).

Keymer et al. (2017) supports this assumption by showing that reduced plant fatty acid biosynthesis impairs pathogenic fungal infection, further indicating that host-to-parasite lipid transfer might be an important node during the infection process. However, an explanation of how fatty acids are transferred to powdery mildews must await further molecular experiments using fungal transgenic systems (Martinez-Cruz et al., 2017).

### Obligate Biotrophic Niches Are Potential Causes of Powdery Mildews’ Adaptive Evolution

**FIGURE 7:**
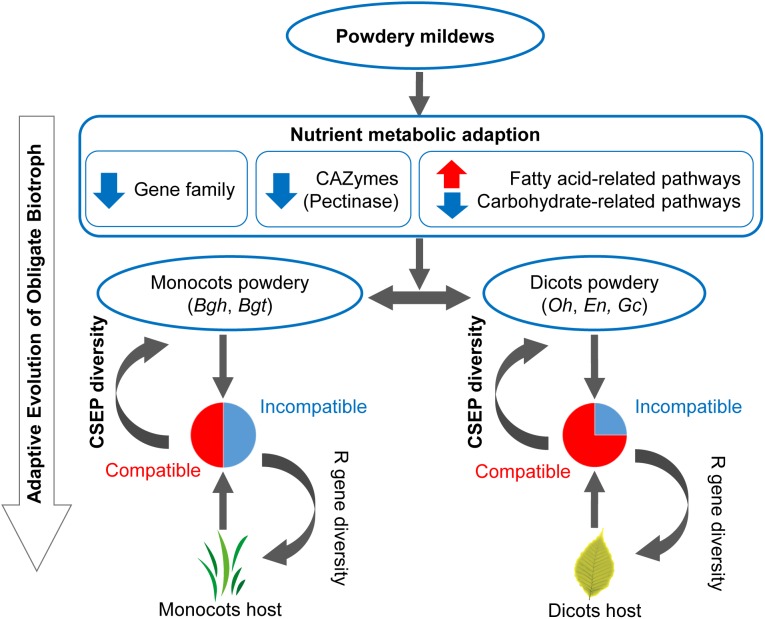
Evolutionary model of inefficient carbohydrate metabolism pathway and diverse CSEPs enables an adaptation to an obligate biotrophic lifestyle. Red arrows indicate gene family/pathway expansion. Blue arrows indicate gene family/pathway contraction. Pie plots indicate common incompatible interactions between monocot powdery mildews and monocot plants and infrequent incompatible interactions between dicot powdery mildews and dicot plants. CSEP, candidate secreted effector protein. R gene, plants’ resistance gene.

In this work, comparative analysis of 15 microbial genomes revealed the following potential evolutionary pattern of powdery mildews’ adaptation to obligate biotrophic niches. First, contraction of carbohydrate-active enzymes offers powdery mildews a possibility to use fatty acids as an alternative carbon source (Figure 2C). Then the strong positive selection on small CSEP groups in monocot, than in dicot powdery mildews lead to diversification of powdery mildew CSEPs (Figure 6). These two factors—the change in nutrient metabolism pathways and rapid evolution of CSEPs—provide evolutionary features for powdery mildews (Figure 7).

## Conclusion

In conclusion, we have presented integrative genomic clues that provide insights into the contracted carbohydrate metabolism associated with the obligate biotrophic lifestyle. Our findings show CSEP divergences between different pathogen lineages and support the assertion that CSEPs represent species-specific adaptations.

## Materials and Methods

### Biological Materials

The powdery mildew pathogen *Oidium heveae* (strain HO-73) was single-spore purified and maintained on rubber tree Reyan 7-33-97 grafted plants in a chamber at Hainan University, Haikou, China (Li et al., 2016). The plants were incubated in a growth chamber at 22°C with approximately 70% humidity and a 16-h light/8-h dark cycle. For inoculation, conidia were dusted from infected leaves to healthy, tender leaves. Infected leaves were shaken 24 h before inoculation to allow the formation of fresh conidial spores. Conidia on the surface were collected by aspiration attached to a vacuum port. The conidia were then transferred to a sterile centrifuge tube and all samples were immediately frozen in liquid nitrogen and stored at -80°C until total DNA and RNA extraction.

### Genome Sequencing and Assembly

Genomic DNA, extracted from *O. heveae* conidia and mycelia using the OMEGA Fungal DNA Kit (Norcross, GA, USA), was used to sequence the genome using Illumina HiSeq technology (150-bp, 300-bp, and 800-bp paired-end reads and 3 k mate-pair reads). Insert size and library quality were confirmed using an Agilent 2100 Bioanalyzer before sequencing. We obtained a total of 107 million pair-end reads and 111 million mate-pair reads then assessed read quality with FastQC (Babraham Bioinformatics, Cambridge, England). We mapped the *O. heveae* reads to the rubber genome (Tang et al., 2016) and excluded the reads of successful alignment. We used SOAPdenovo2 with multiple *k*-mer sizes to achieve optimal assembly results with the highest assembly quality based on the N50 metric (Luo et al., 2012). GapCloser software filled in the remaining local, inner gaps, thus completing the assembly. Gene space reliability was assessed by the Core Eukaryotic Genes Mapping Approach (CEGMA) (Parra et al., 2007).

### Genome Size and Repeat Predictions

We first estimated the repeat content size by counting *k*-mer occurrences using Jellyfish (*k* = 17 bp) (Marçais and Kingsford, 2011). Since many assembled powdery mildew genomes are composed of repeat sequences, we then used RepeatModeler to perform *ab initio* repeat family sequence predictions and all repeat families were compared with Repbase sequences for classification (Bao et al., 2015). A consensus repeat element library identified for each species was fed into the downstream annotation pipeline.

### Gene Prediction

For *O. heveae* gene predictions, we applied the MAKER2 annotation pipeline (Holt and Yandell, 2011) and used *ab initio* gene predictor tools such as SNAP (Korf, 2004), Augustus (Stanke and Waack, 2003), and GeneMark-ES (Ter-Hovhannisyan et al., 2008) to predict gene models in the genome. To improve gene model prediction quality, we performed RNA-sequencing at four infection time points [0 h post-inoculation (hpi), 24 hpi, 3 days post-inoculation (dpi), and 30 dpi] using TRIzol Reagent (Thermo Scientific, Waltham, MA, United States) for total RNA extraction from conidia and hyphae. Then, transcripts were *de novo* assembled using a genome-guided strategy by Trinity (Grabherr et al., 2011). We then used MAKER2 to merge all *ab initio* predictions; assembled RNA-seq transcripts; and identified proteins from *Blumeria graminis* f. sp. *hordei*, *Blumeria graminis* f. sp. *tritici*, and *Erysiphe necator* to generate a set of predicted gene models. We identified tRNAs by using tRNAscan-SE and rRNAs by using Barrnap.

### Functional Annotation

We annotated all coding protein genes from 15 microorganisms. Gene functions were assigned according to the best matches of BLASTP alignment against the NCBI non-redundant protein sequence database (*E*-value < 1.0 × 10^-5^). Using Blast2GO (Götz et al., 2008) and the KEGG Orthology-based Annotation System (KOBAS), we completed Gene Ontology (GO) and Kyoto Encyclopedia of Genes and Genomes (KEGG) annotations then searched Pfam (*P*-value < 1 × 10^-5^) (Finn et al., 2014) to determine protein domains. For secretome prediction, proteins were analyzed in SignalP v4.1 to predict secretory signal peptides (Petersen et al., 2011), then mature proteins were identified by the TMHMM v2.0 program (Moller et al., 2001). Only proteins possessing N-terminal signal peptides and no transmembrane domains within the mature protein were selected and only secretomes were used as input genes to identify carbohydrate-active enzymes and proteases using the dbCAN HMM-based classification system (*E*-value < 1.0 × 10^-4^) for carbohydrate-active enzyme annotation (Yin et al., 2012). To identify secreted protease genes, the MEROPS database 10.0 was searched for predicted secreted proteins (*E*-value < 1 × 10^-5^) (Rawlings et al., 2014). Transporters were identified in BLAST searches using the Transporter Collection Database (*E*-value < 1.0 × 10^-5^) (Saier et al., 2016) and BLASTP queries using the Pathogen-Host Interaction database (PHI-base) with a threshold (*E*-value < 1 × 10^-5^) searched for potential microorganismal, pathogenicity-related proteins (Urban et al., 2015). All the carbohydrate-active enzymes (CAZymes), protease, transporter, and PHI classification gene counts were used as input for principal component analysis.

### Gene Family Cluster and Phylogenetic Analysis

We used the OrthoMCL v2.0.9 package to identify gene families for the 15 species (Li et al., 2003), determining species-specific gene families based on the presence or absence of genes for a given species. All orthologous families for each lifestyle were calculated, mapped, and illustrated using a Venn diagram. We generated 19,876 family groups, from which 287 single-copy orthologous genes were first aligned using MAFFT v7.299b and then concatenated (Katoh and Standley, 2013). Resultant multiple alignments were then analyzed with Gblocks v0.91b using default parameters to select conserved regions. The best amino acid substitution model (LG+I+G model) was chosen using ProtTest v3.4.2 (Darriba et al., 2011) then a phylogenetic tree, based on the maximum likelihood method with 1,000 bootstrap replicates, was constructed using RAxML v8.2 (Stamatakis, 2014). We estimated divergence times for each node in the phylogenetic tree using the MCMCtree program from the PAML v4.9b package (Yang, 2007), with four reported divergence times used for calibration. The first divergence time [*Oomycota* and Fungi between 1.502 and 2.035 billion years ago (Bya)] formed the root. The second divergence time was for *Basidiomycota* and *Ascomycota* [approximately 603–844 million years ago (Mya)] and the next, for *Saccharomycotina* and *Pezizomycotina*, was approximately 460–726 Mya. The divergence time for *Erysiphales* was from 58 to 70 Mya (Takamatsu, 2013) and the last time, from 5.2 to 7.4 Mya, was for cereal powdery mildews (Wicker et al., 2013). Then, divergence times were retrieved from the TimeTree database (Xia et al., 2017) and the final trees were built with FigTree.

### Gene Family Evolution

We investigated the dynamic expansion and contraction of gene families using the CAFÉ program with the ultrametric tree obtained from MCMCtree (Han et al., 2013). Overall, we inferred that 19,876 gene families, from the 15 species, were present in the most common recent ancestor. To provide an accurate birth/death parameter over the tree (lambda), we corrected potential genome assembly and annotation errors using the “errormodel” command in the Python script caferror.py, using *P* < 0.05 for significantly changed gene families. Further KEGG enrichment analysis of expanded or contracted families was conducted using KOBAS with Fisher’s exact test.

### MACG Identification

We identified missing ascomycete core genes (MACGs) from orthologous gene families absent in the powdery mildews but present in the phytopathogens *Colletotrichum higginsianum*, *Magnaporthe oryzae*, *Fusarium graminearum*, *Sclerotinia sclerotiorum*, and *Botrytis cinerea.*

### Pairwise Gene *K*a/*K*s

To elucidate divergent evolution among powdery mildews, we calculated pairwise synonymous and non-synonymous substitution (*K*a/*K*s) rates for single-copy orthologous genes. We generated 4,136 single-copy orthologous groups shared by four powdery mildews, 836 single-copy groups shared by monocot powdery mildews, and 404 single-copy groups shared by dicot powdery mildews. The sequences from each family were aligned using MAFFT and RevTrans based on the protein sequences and back-translated into codon alignments. The KaKs_Calculator 2.0 tool was used to calculate pairwise *K*a/*K*s values (Wang et al., 2010) with Fisher’s exact test assessing the significance level of the selection.

### CSEP Prediction and Analysis

CSEPs were identified by similar criteria as described previously (Pedersen et al., 2012). The secreted proteins were identified by SignalP v4.1 and TMHMM v2.0. We used the secreted proteins as the query for BLASTP searches against the NCBI non-redundant database (*E*-value < 1.0 × 10^-5^). The secreted proteins that had no similarity to other proteins, except for hits to powdery mildews, were identified as CSEP. Finally, to understand the cell localization, TargetP (Emanuelsson et al., 2007) and big-PI fungal predictor (Eisenhaber et al., 1998) were also integrated into pipeline (Supplementary Figure S9). We used MEGA5 to generate a MUSCLE sequence alignment, and bootstrap consensus and neighbor-joining trees (Tamura et al., 2011). To compare the non-classically secreted effector repertoire, we used SignalP and SecretomeP to identify non-classically secreted effectors (Supplementary Figure S9).

We conducted InterProScan5 analysis to identify functional domains (Jones P. et al., 2014), improving identification by using template 1fus.1.A (Vassylyev et al., 1993), which was obtained from the PDB website (Berman et al., 2000). We used SWISS-MODEL’s alignment mode to complete homology modeling (Waterhouse et al., 2018) and visually inspected each model, highlighting the residues using Open-Source PyMol^2^. Then we used the site model and branch-site model from the PAML program (Yang, 2007) to estimate the positive site. The sequence logos were created using WebLogo (Crooks et al., 2004), marking the positive sites, and disulfide bonds were predicted with Disulfind (Ceroni et al., 2006).

## Accession Numbers

The *O. heveae* genome and RNA-seq sequences have been deposited in GenBank/DDBJ/EMBL under the accession codes of QVIK00000000 and SRP158299, respectively

## Data Availability Statement

Coding sequence set of *O. heveae*, and other datasets generated for this study are included in the manuscript and in the Supplementary Files.

## Author Contributions

PL and WM conceptualized the research program. PL, WM, and XW coordinated the project. PL, QH, WL, and CL contributed to fungal and plant materials and extracted DNA and RNA. PL, FX, SL, and FZ performed genome annotation and comparative genome analysis. SJ, PL, and JY conducted statistical analysis and phylogenetic analysis. PL, XW, WM, and FZ wrote and revised the manuscript.

## Conflict of Interest Statement

The authors declare that the research was conducted in the absence of any commercial or financial relationships that could be construed as a potential conflict of interest.

## Footnotes

1 https://genome.jgi.doe.gov/Golci1/Golci1.home.html

2 http://www.pymol.org
