# Corrigendum: Powdery Mildews Are Characterized by Contracted Carbohydrate Metabolism and Diverse Effectors to Adapt to Obligate Biotrophic Lifestyle

**DOI:** 10.3389/fmicb.2019.00001

**Published:** 2019-01-23

**Authors:** Peng Liang, Songyu Liu, Feng Xu, Shuqin Jiang, Jun Yan, Qiguang He, Wenbo Liu, Chunhua Lin, Fucong Zheng, Xiangfeng Wang, Weiguo Miao

**Affiliations:** ^1^College of Plant Protection, Hainan University, Haikou, China; ^2^Key Laboratory of Green Prevention and Control of Tropical Plant Diseases and Pests (Hainan University), Ministry of Education, Haikou, China; ^3^Department of Crop Genomics and Bioinformatics, College of Agronomy and Biotechnology, National Maize Improvement Center of China, China Agricultural University, Beijing, China; ^4^State Key Laboratory of Agrobiotechnology, College of Biological Sciences, China Agricultural University, Beijing, China

**Keywords:** *Erysiphales*, *Oidium heveae*, genome, gene family contraction, fatty acids, CSEPs, positive selection, adaptive evolution

The order of correspondence authors was incorrectly provided as “Xiangfeng Wang, sysbio@cau.edu.cn” and “Weiguo Miao, miao@hainu.edu.cn” in the Correspondence section. The correct order should be “Weiguo Miao, miao@hainu.edu.cn” and “Xiangfeng Wang, sysbio@cau.edu.cn.”

Also, in the published article, the RNA-seq sequence number SRP158299 was removed by mistake from the “Accession Numbers” section. The correct Accession Numbers statement should read as follows:

“The *O. heveae* genome and RNA-seq sequences have been deposited in GenBank/DDBJ/EMBL under the accession codes of QVIK00000000 and SRP158299, respectively.”

The authors apologize for these errors and state that they do not change the scientific conclusions of the article in any way. The original article has been updated.

