# Supplementary material for: Powdery Mildews Are Characterized by Contracted Carbohydrate Metabolism and Diverse Effectors to Adapt to Obligate Biotrophic Lifestyle
Source: Front Microbiol. 2018 Dec 18;9:3160. doi: 10.3389/fmicb.2018.03160 (PMC6305591; doi:10.3389/fmicb.2018.03160)
Supplement: Supplementary file 3 [file Data_Sheet_2.docx]

Supplementary Material

# Powdery mildews are characterized by contracted carbohydrate metabolism and diverse effectors to adapt to obligate biotrophic niches

**Peng Liang^1, 2, 3^, Songyu Liu^4^, Feng Xu^3^, Shuqin Jiang^3^, Jun Yan^3^, Qiguang He^1, 2^, Wenbo Liu^1, 2^, Chunhua Lin^1, 2^, Xiangfeng Wang^3, *^ and Weiguo Miao^1, 2, *^**

*** Correspondence:** Weiguo Miao: miao@hainu.edu.cn, Xiangfeng Wang: sysbio@cau.edu.cn

**Supplementary Figures**

**Figure S1.** Morphological observations of different key developmental stages in powdery mildews.

**Figure S2.** K-mer depth distribution plot of Illumina reads.

**Figure S3.** Comparison of CAZymes, protease, transporter, and PHI classifications among microorganisms with different lifestyles.

**Figure S4.** Comparison of pectinase family members among microorganisms with different lifestyles.

**Figure S5.** Loss of pectinase in the pentose and glucuronate interconversions pathway in powdery mildews genomes.

**Figure S6.** Comparison of the fatty acid biosynthesis pathway among powdery mildews and other phytopathogens.

**Figure S7.** Comparison of the fatty acid elongation pathway among powdery mildews and other phytopathogens.

**Figure S8.** Comparison of the citrate (TCA) cycle pathway among powdery mildews and other phytopathogens.

**Figure S9.** CSEP identification pipeline.

**Figure S10.** CSEP phylogenetic tree in *Oidium heveae*.

**Figure S11.** Sequence logo of CSEPs in orthologous group 1.


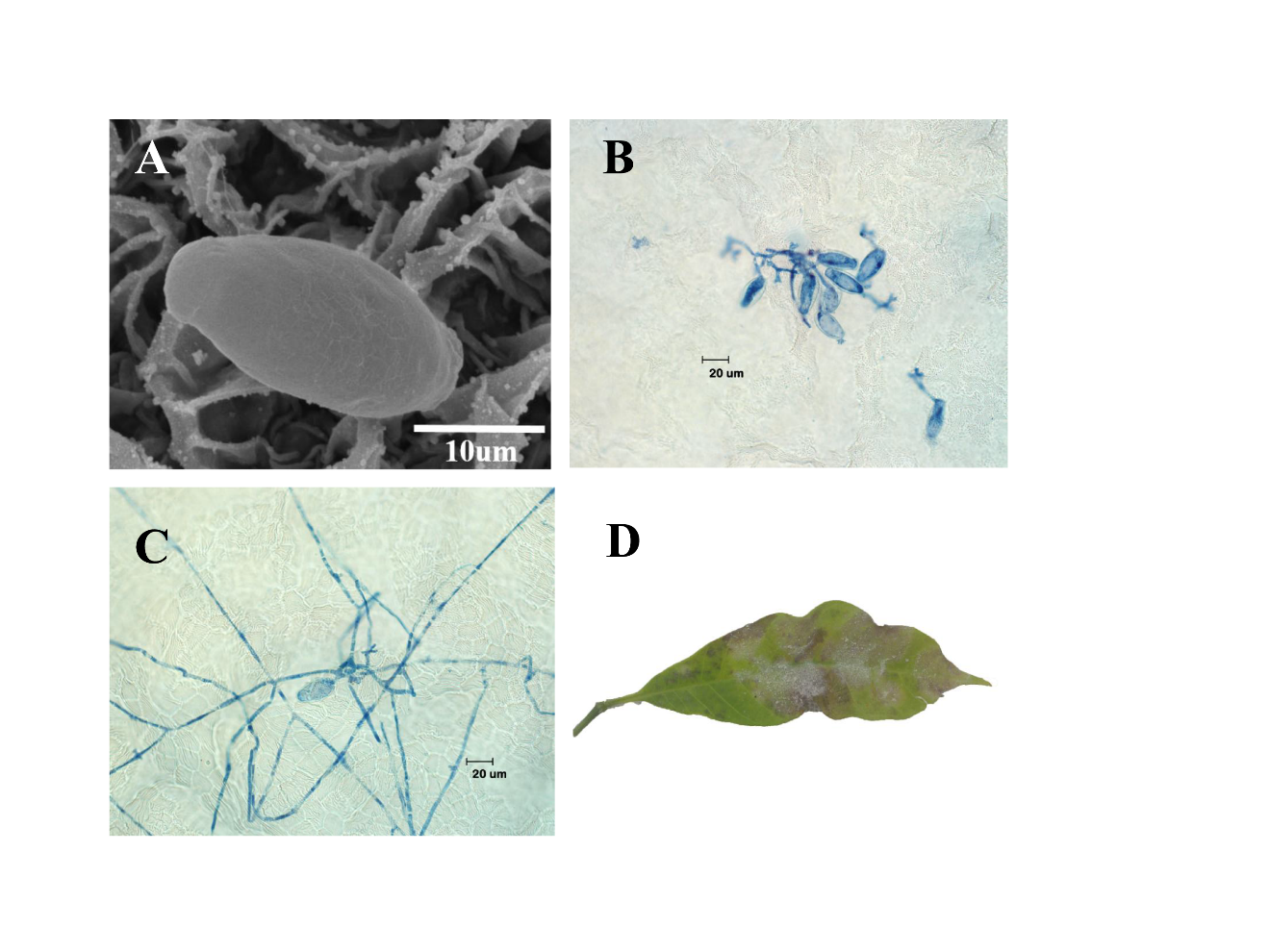


**Figure S1.** Morphological observations of different key developmental stages in powdery mildew. (A) Conidium prior to germination on leaf surface of rubber tree. Bar=10um. (B) Conidia germinated on host and differentiated appressoria after 24 hours post inoculation (24hpi). Bar=20um. (C) Hyphae ramify across the leaf surface of rubber tree after 3 days post inoculation (3dpi). Bar=20um. (D) Infected leaf with curling and symptom after 30 days post inoculation (30dpi).


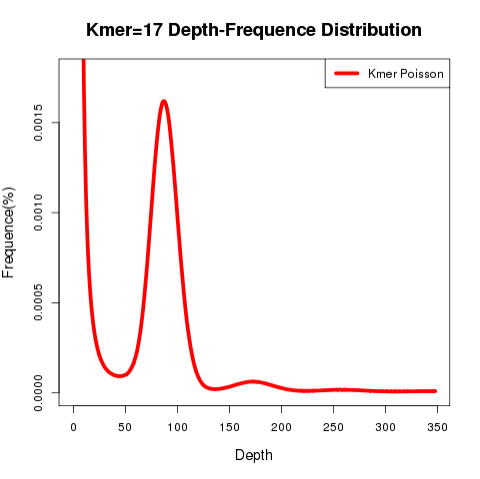


**Figure S2.** K-mer depth distribution plot of Illumina reads.


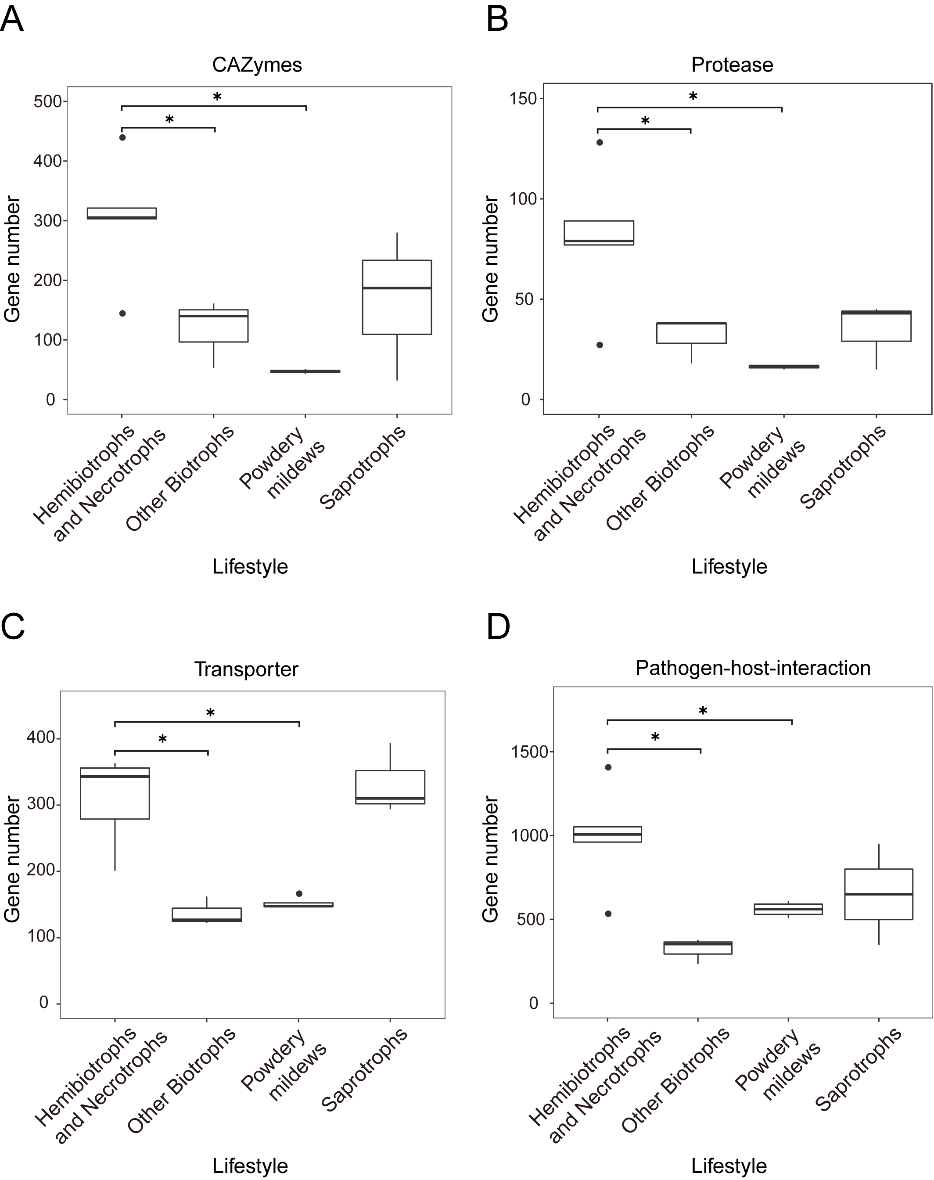


**Figure S3.** Comparison of CAZymes, protease, transporter, and PHI classifications among microorganisms with different lifestyles. (A) The number of CAZymes were less in powdery and other biotrophs than in hemibiotrophs and necrotrophs. (B) The number of protease were less in powdery than in hemibiotrophs and necrotrophs. (C) The number of transporter were less in other biotrophs than in hemibiotrophs and necrotrophs. (D) The number of PHI were less in other biotrophs than in hemibiotrophs and necrotrophs. Asterisks represent significant difference determined by Student’s t-test (* *P* < 0.05).


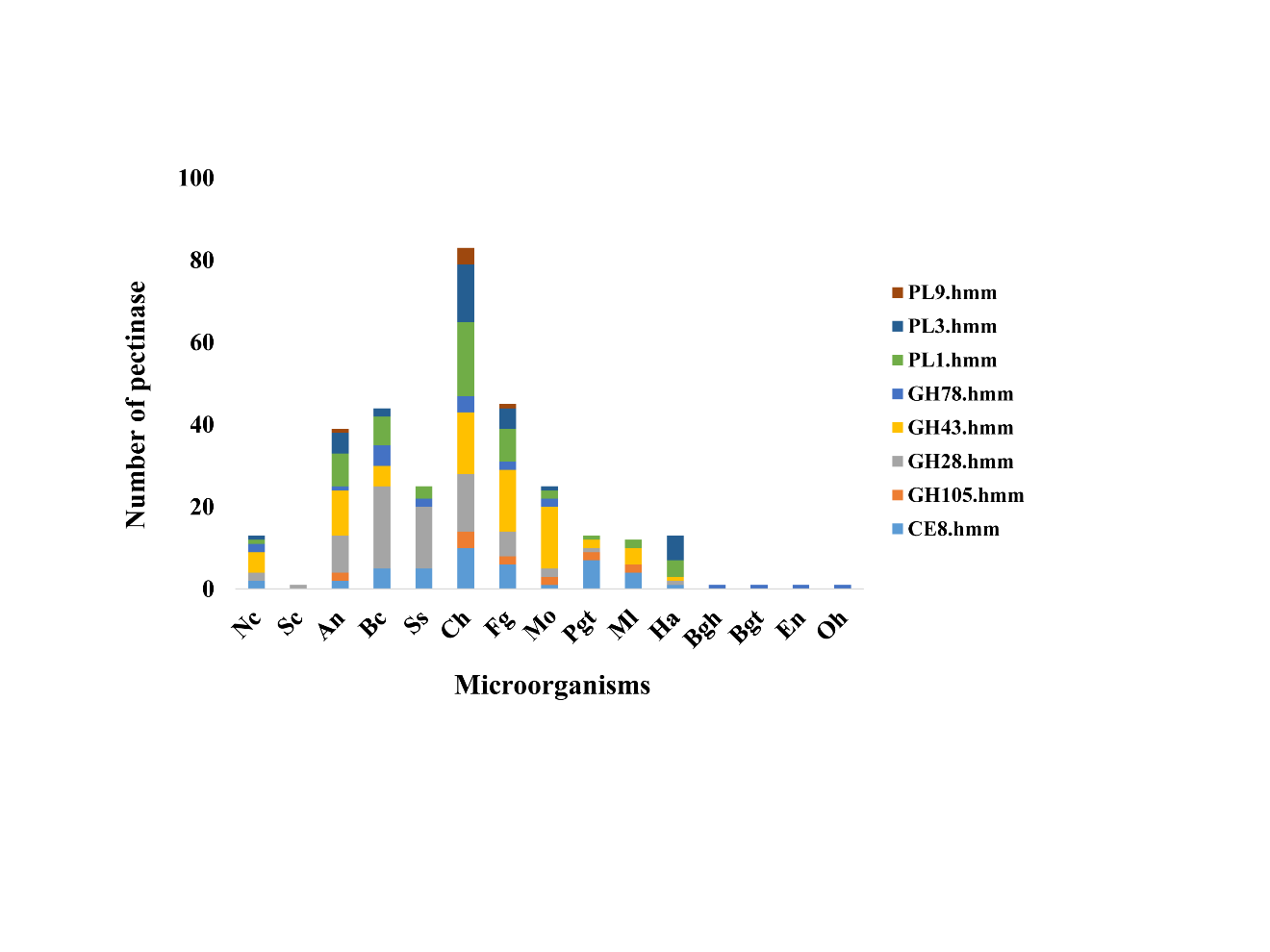


**Figure S4.** Comparison of pectinase family members among microorganisms with different lifestyles. The height of each segment in the stacked bars represents the predicted number of pectinase assigned to each of the superfamilies of CAZyme, i.e. glycoside hydrolases (GHs), polysaccharide lyases (PLs) and carbohydrate esterases (CEs).


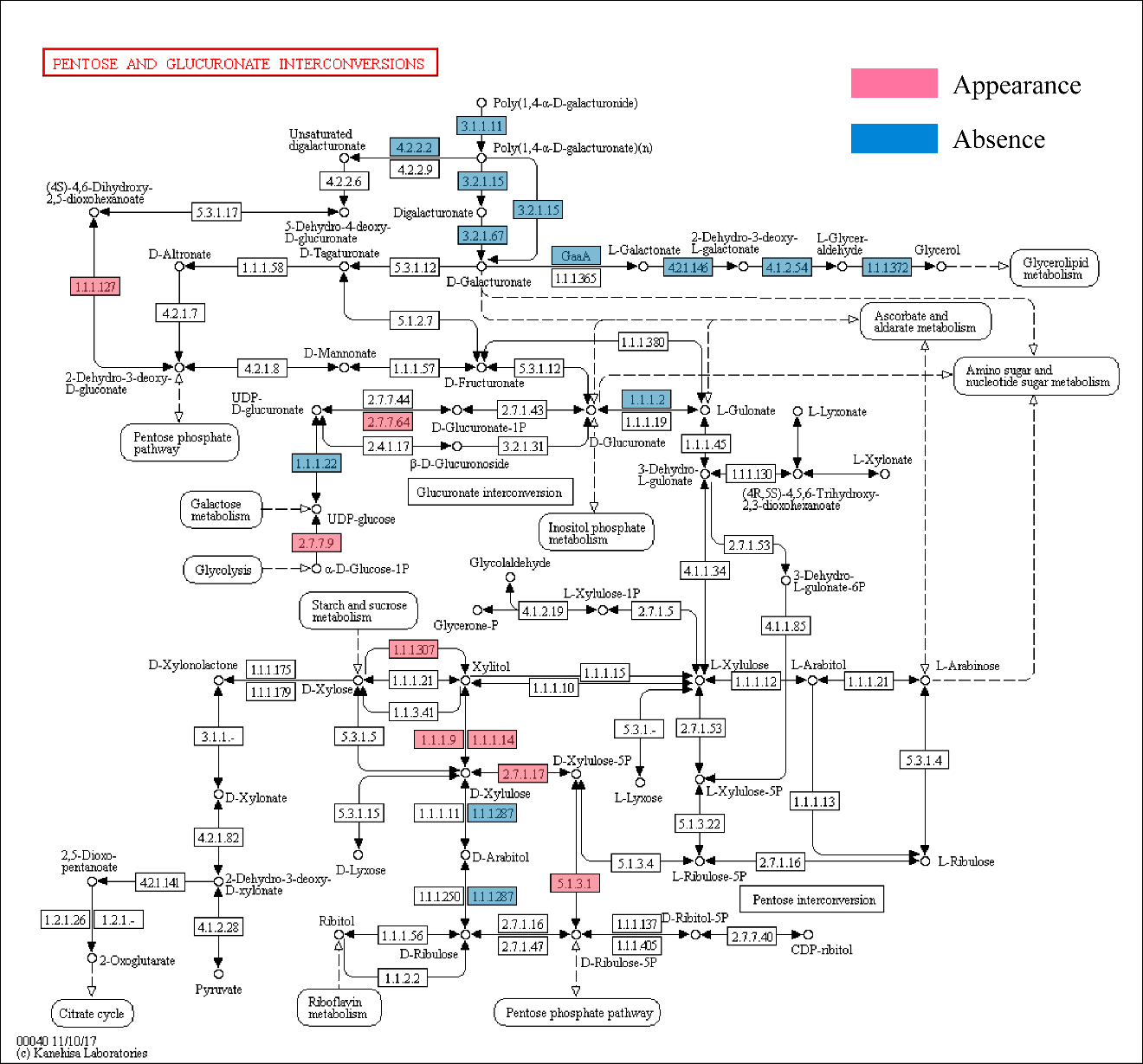


**Figure S5.** Loss of pectinase in the pentose and glucuronate interconversions pathway in powdery mildews genomes. Red box indicates appearance of the enzyme in powdery mildews and other *ascomycete* phytopathogens, blue box indicates absence of the enzyme in powdery mildews while the enzymes were appeared in other *ascomycete* phytopathogens, the same occur in below figures.


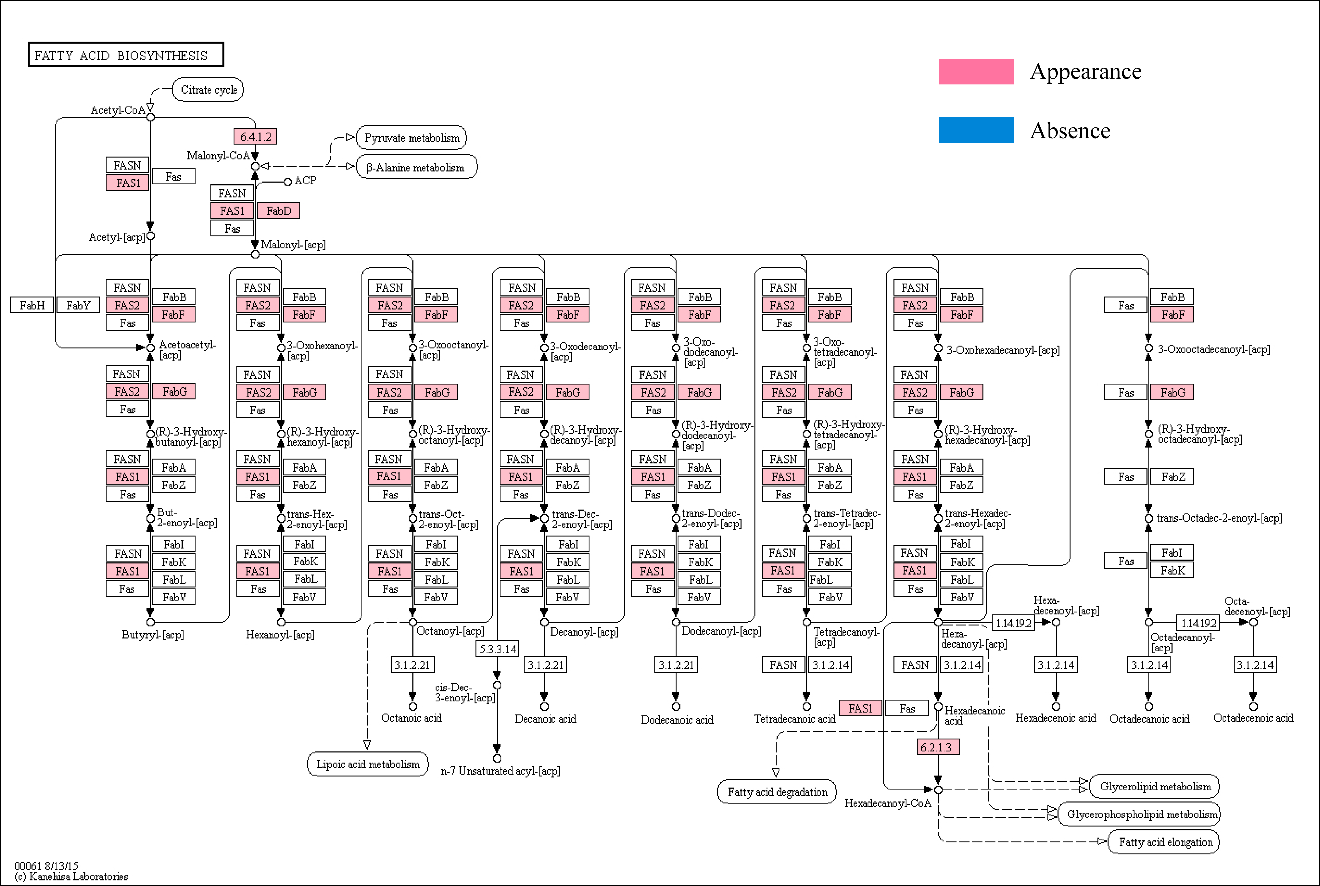


**Figure S6.** Comparison of the fatty acid biosynthesis pathway among powdery mildews and other phytopathogens.


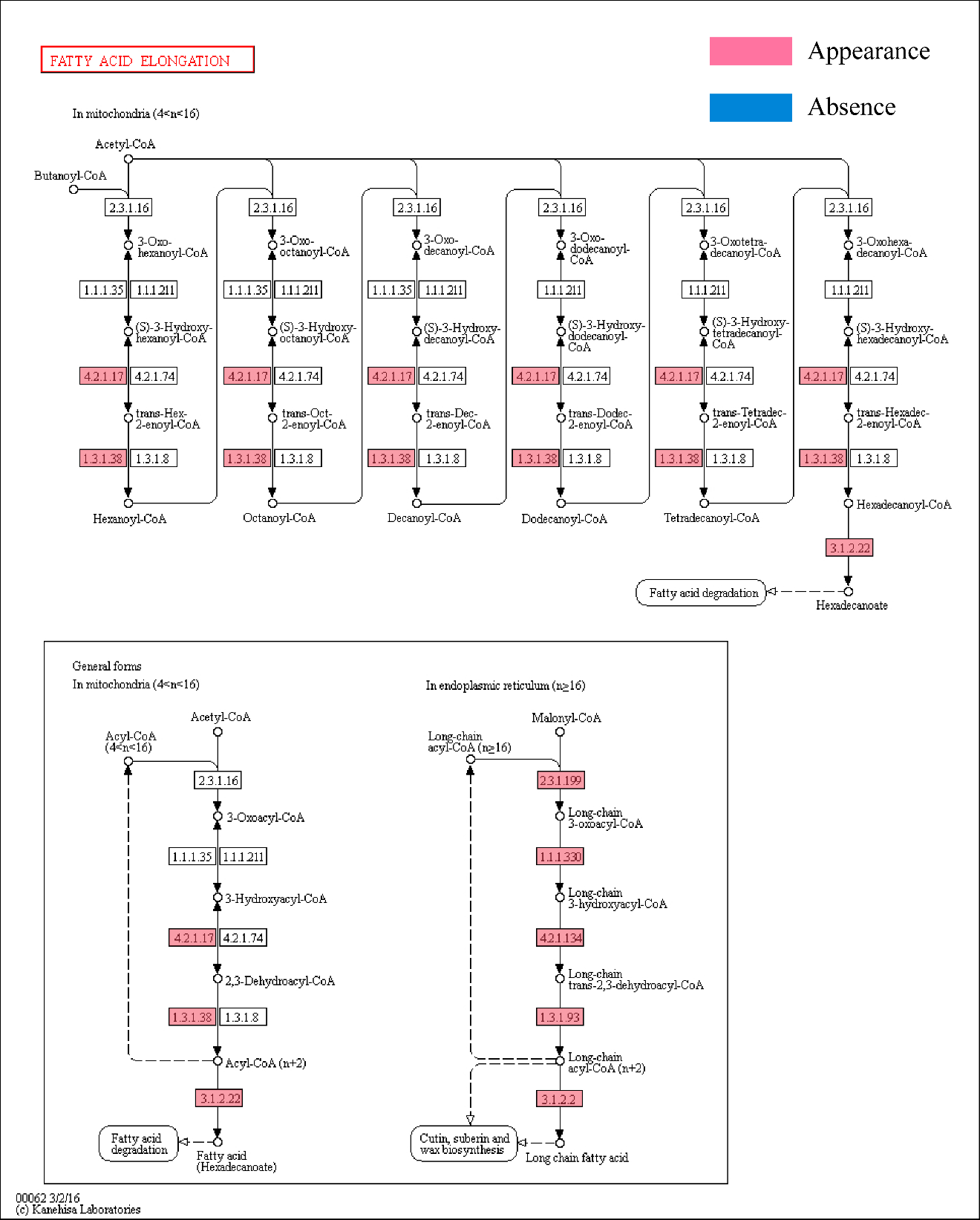


**Figure S7.** Comparison of the fatty acid elongation pathway among powdery mildews and other phytopathogens.


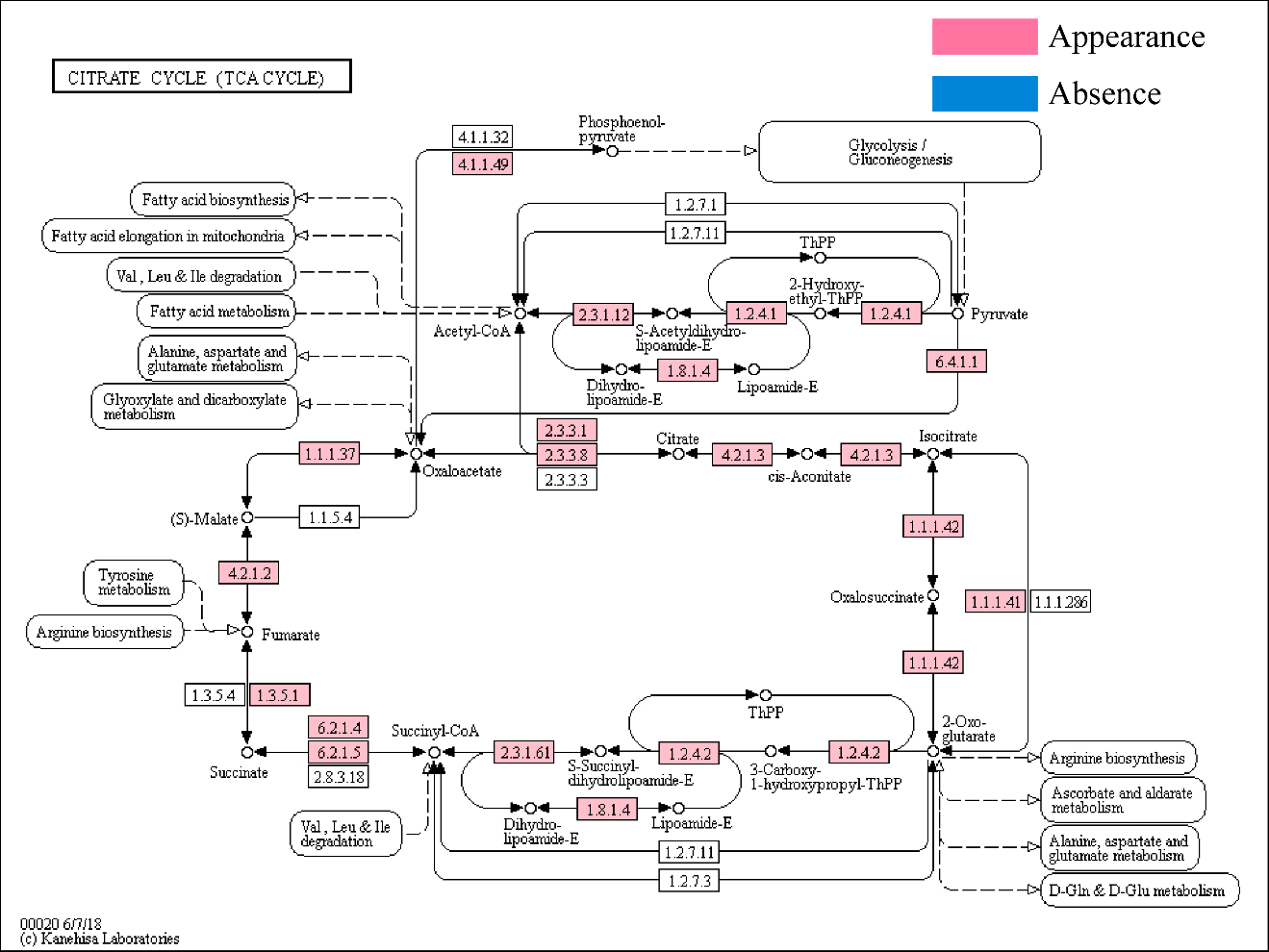


**Figure S8.** Comparison of the citrate (TCA) cycle pathway among powdery mildews and other phytopathogens.


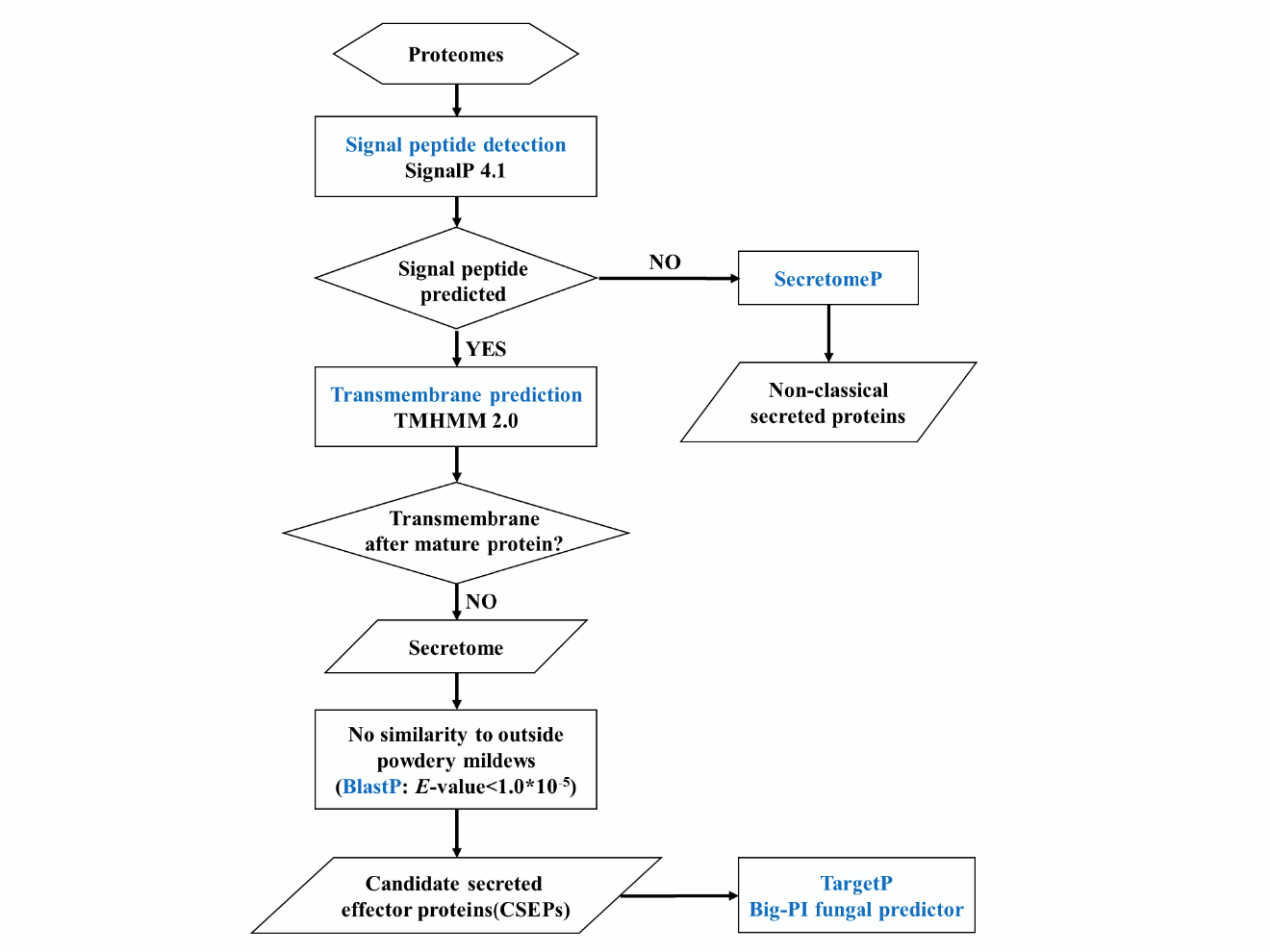


**Figure S9.** CSEP identification pipeline.


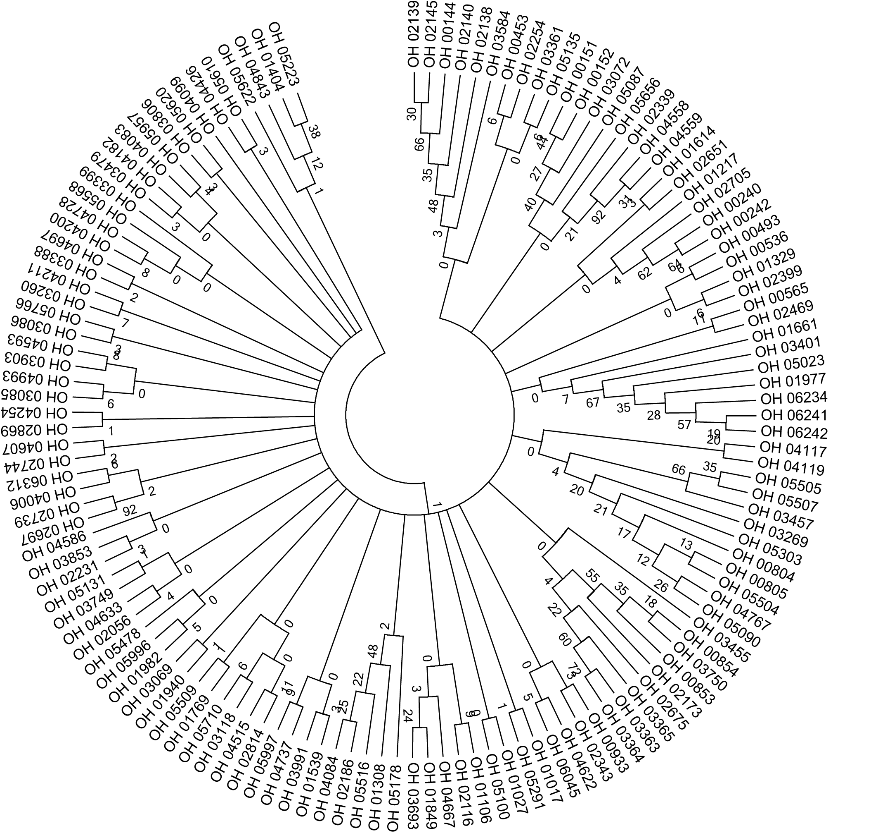


**Figure S10.** CSEP phylogenetic tree in *Oidium heveae*.


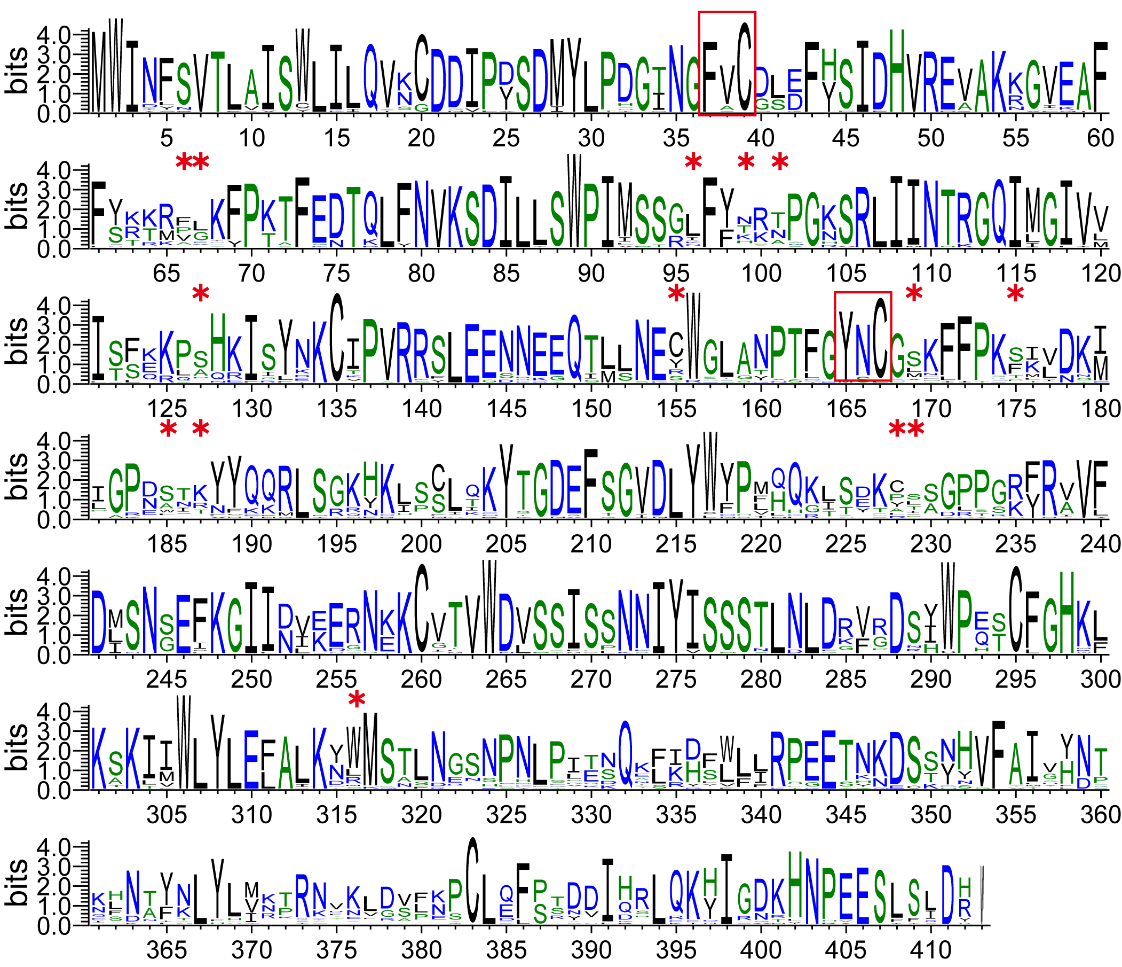


**Figure S11.** Sequence logo of CSEPs in orthologous group 1. The asterisk indicates positive site of genes. The red frames indicate [Y/W/F]xC motif. The bigger letter indicates the higher similarity in the site.
